# Nicotinic Acid-Mediated Activation of Both Membrane and Nuclear Receptors towards Therapeutic Glucocorticoid Mimetics for Treating Multiple Sclerosis

**DOI:** 10.1155/2009/853707

**Published:** 2009-05-17

**Authors:** W. Todd Penberthy

**Affiliations:** Department of Molecular Genetics, Biochemistry, and Microbiology, University of Cincinnati, 231 Albert Sabin Way P.O. Box 670524, 2938 CVC Mail Loc-0524, Cincinnati, Ohio 45237, USA

## Abstract

Acute attacks of multiple sclerosis (MS) are most commonly treated with glucocorticoids, which can provide life-saving albeit only temporary symptomatic relief. The mechanism of action (MOA) is now known to involve induction of indoleamine 2,3-dioxygenase (IDO) and interleukin-10 (IL-10), where IL-10 requires subsequent heme oxygenase-1 (HMOX-1) induction. Ectopic expression studies reveal that even small changes in expression of IDO, HMOX-1, or mitochondrial superoxide dismutase (SOD2) can prevent demyelination in experimental autoimmune encephalomyelitis (EAE) animal models of MS. An alternative to glucocorticoids is needed for a long-term treatment of MS. A distinctly short list of endogenous activators of both membrane G-protein-coupled receptors and nuclear peroxisome proliferating antigen receptors (PPARs) demonstrably ameliorate EAE pathogenesis by MOAs resembling that of glucocorticoids. These dual activators and potential MS therapeutics include endocannabinoids and the prostaglandin 15-deoxy-Δ^12,14^-PGJ_2_. Nicotinamide profoundly ameliorates and prevents autoimmune-mediated demyelination in EAE via maintaining levels of nicotinamide adenine dinucleotide (NAD), without activating PPAR nor any G-protein-coupled receptor. By comparison, nicotinic acid provides even greater levels of NAD than nicotinamide in many tissues, while additionally activating the PPAR*γ*-dependent pathway already shown to provide relief in animal models of MS after activation of GPR109a/HM74a. Thus nicotinic acid is uniquely suited for providing therapeutic relief in MS. However nicotinic acid is unexamined in MS research. Nicotinic acid penetrates the blood brain barrier, cures pellagric dementia, has been used for over 50 years clinically without toxicity, and raises HDL concentrations to a greater degree than any pharmaceutical, thus providing unparalleled benefits against lipodystrophy. Summary analysis reveals that the expected therapeutic benefits of high-dose nicotinic acid administration far outweigh any known adverse risks in consideration for the treatment of multiple sclerosis.

## 1. Multiple Sclerosis Treatment and Functional Transcriptomics

Multiple sclerosis (MS) is the most common demyelinating disease of the central nervous system. It is a progressive disease with no known cure. MS results in scarring of CNS tissues termed sclerosis due to autoimmune-mediated attack of myelin-forming oligodendrocytes and/or myelin sheaths by autoreactive T cells. The disease affects more than 2.5 million people worldwide; 30% of MS patients eventually develop paralysis and become wheelchair bound for the rest of their lives [1, 2].

Glucocorticoids are the primary pharmacotherapeutic approach used to provide relief from acute attacks of MS and are the most commonly prescribed drugs in the world for treating autoimmune disorders in general (for a review [3]). While we are far away from a comprehensive picture of how glucocorticoids control neuroinflammation, recent studies have revealed central roles for indoleamine 2,3-dioxygenase (IDO; [4, 5]) and interleukin-10 (IL-10; [6]). The importance of IDO in estrogen-mediated suppression of EAE pathogenesis was demonstrated previously [7]. IL-10 signaling is required for patients to respond to glucocorticoid treatment [6], while IL-10-mediated induction of heme-oxygenase-1 (HMOX-1) is required for IL-10 to exert its anti-inflammatory activity [8]. Increases in HMOX-1 have recently been shown to reverse paralysis and prevent relapse in animal models of MS [9]. Thus, by boosting IDO and IL-10 or HMOX-1, greater therapeutic benefit against autoimmune demyelinating disease may be achieved. Alternative clinical MS therapeutics have also been shown to work via induction of IL-10 or IDO. This includes the amino acid copolymer glatiramer acetate [10] or interferons [11–18], respectively. It seems difficult, however, to replicate the endogenous anatomically localized cellular production of interferon via pharmacological approaches. Interferon treatment is associated with complications, some of which can be quite serious [19]. Ultimately, no pharmacological agent has been proven to be clinically effective in patients during the progressive stages of MS [20].

The most common animal model of MS involves injection of myelin sheath-associated epitopes into mammals, which results in a dose dependent experimental autoimmune encephalomyelitis (EAE) (for a review see [21]). Lymphocyte-mediated demyelination proceeds around blood vessels of the CNS, leading to autoimmune-mediated paralysis typically 10 days to three weeks postinjection. EAE has been successfully used in the development of clinical therapeutics [22]). Studies examining the pharmacology of the endogenous PPAR*γ* activators have consistently revealed that the endogenous molecule 15-deoxy-Δ^12,14^-prostaglandin J(2) (15d-PGJ_2_) can ameliorate the MS clinical symptoms in EAE [23–27]. 15d-PGJ_2_ is the most known potent endogenous activator of PPARs identified to date [25]. By focusing on endogenous molecules, we can better understand the inherent mechanisms by which the body maintains good health. This includes the endogenous PPAR activators 15d-PGJ_2_, two endocannabinoids (anandamide and 2-arachidonoylglycerol; 2-AG), and nicotinic acid, which stimulates the localized production of 15d-PGJ_2_ restricted to professional antigen presenting cells.

PPAR activators are well known for their ability to correct dyslipidemia [27]. Nicotinic acid indirectly activates PPAR [28, 29] and corrects dyslipidemia, raising high-density lipoprotein (HDL) concentrations to a greater degree than any known pharmaceutical [30]. The other most common nicotinamide adenine dinucleotide (NAD) precursor, nicotinamide, provides dramatic protection against demyelination and improves behavioral deficits in EAE [31]. Unlike nicotinic acid, nicotinamide provides little benefit against dyslipidemia since it does not bind to the high-affinity nicotinic acid G-protein-coupled receptor, and therefore nicotinamide does not activate PPAR*γ*. By contrast, nicotinic acid has not been systematically examined in animal models of MS or in the clinic.

Either nicotinamide or nicotinic acid can serve essential functions as a dietary precursor to NAD in the cell. Nicotinic acid is the most preferred substrate in evolution based on metabolite [32, 33] and comparative genomic studies examined to date (presentation by Mathias Ziegler at PARP 2008 meeting, Tucson, Ariz, USA). In glia, nicotinic acid provides greater levels of NAD biosynthesis per mole than nicotinamide or tryptophan by 200- and 500-fold, respectively [34]. Furthermore, neurons appear distinctively inefficient in their ability to convert dietary NAD precursors to NAD [35].

Accordingly, supraphysiological elevations of NAD are known to exert tremendous neurotrophic activity. When a neuron is cut with a razor blade, degeneration of axons occurs typically within one day. Genetic triplication of the nuclear generating enzyme nicotinamide adenine mononucleotidyl transferase-1 (NMNAT1) however delays neurodegeneration for 2-3 weeks after excision [36, 37]! This dramatic effect is mediated via NAD-dependent activation of SIRT-1. Functioning as a NAD-dependent histone deacetylase, SIRT-1 exerts global changes in the transcriptome that mimic caloric restriction (for a review of this process see [38]). Direct SIRT-1-mediated deacetylation of the liver X receptor (LXR) leads to activation of LXR with effects on lipid homeostasis and ABCA1 gene regulation [39]. Similarly, SIRT-1 directly deacetylates and activates peroxisome proliferator-activated receptor-gamma, coactivator 1 (PGC1*α*). Redistribution of the other nuclear NMNAT1-encoding protein to the cytosol extends the delay in Wallerian degeneration up to seven weeks [40]! While SIRT-2 is present in the cytosol, it has not been determined which pathway is most integral to the cytosolic pathway. Common to both nuclear and cytosolic forms, however, it is clear that NAD is the central to this neurotrophic activity.

Ultimately NAD serves crucial functions as a cofactor in over 200 redox reactions or as a substrate in three categorical enzyme classes: deacetylases (Sirtuins, e.g., SIRT-1), ADP-ribosyl transferases (e.g., PARP-1), and ADP cyclases such as CD38. The two most common MOAs for glia-killing neurons in brain pathologies involve the free-radical generating enzymes NADPH oxidase and iNOS [41]. Both of which can lead to increased PARP-1 activity. Thus pharmacological administration of the NAD precursor nicotinamide provides dramatic protection from the clinical signs of EAE in a dose-dependent manner, preventing behavioral defects, minimizing demyelination, and preventing death [31]. Detailed analysis of the pathways controlling NAD levels and function in the context of multiple sclerosis is reviewed elsewhere [42]. However, nicotinamide treatment does not result in the production of 15d-PGJ_2_ with concomitant activation of PPAR. Collectively these observations suggest even greater benefit against demyelinating disease from nicotinic acid treatment than with nicotinamide. In this review we explore nicotinic acid's effects compared to nicotinamide with focus on describing genes known to be of greatest interest to MS pathogenesis.

Interestingly, high doses of sustained nicotinamide administration (1–4 grams per day) were shown to provide remarkable relief in patients with rheumatoid arthritis in the 1940's [43]. Nicotinamide was then historically supplanted in the treatment of arthritis with the monumental discovery and development of powerful synthetic corticosteroids occurring thereafter.

Today, glucocorticoids remain a common treatment modality in the management of rheumatoid arthritis. However, the ability of nicotinamide to ameliorate the autoimmune aspects of rheumatoid arthritis supports consideration of high-dose nicotinamide treatment in MS.

**Figure 1:**
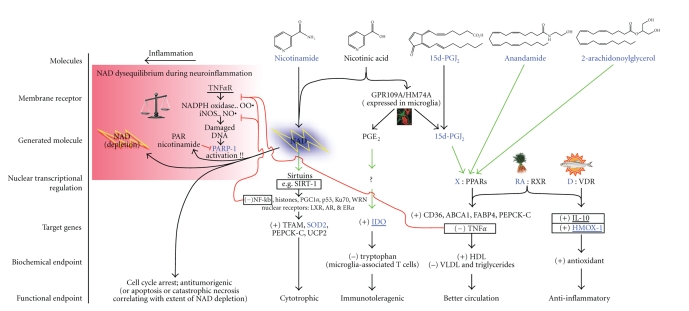
Unique mechanisms of action of nicotinic acid on immune function are shown. Nicotinic acid, but not nicotinamide, binds to the high-affinity nicotinic acid G-protein-coupled receptor HM74a/GPR109a that via calcium influx activates phospholipase A_2_. This ultimately leads to massive production and release of prostaglandins 15d-PGD_2_ and PGD_2_ specifically from professional antigen presenting cells (macrophages, dendritic cells, and likely microglia [55]). Thus, nicotinamide, which also provides NAD, functions in part as a negative control for HM74a-dependent effects in experimentation. PGE_2_ was previously identified as promoting differentiation of plasmacytoid dendritic cells to a T cell toleragenic phenotype via induction of IDO expression and activity [49, 50]. Consequently nicotinic acid may provide a similar T cell toleragenic effect. 15d-PGD_2_ spontaneously degrades to produce 15Δ-PGJ_2_, the most potent endogenous activator of PPAR*γ*. This may be central to the explanation of how nicotinic acid but not nicotinamide corrects dyslipidemia, raising HDL levels higher than any known pharmaceutical including all PPAR*γ* agonists while also lowering LDL, VLDL, and triglycerides [30, 56].

Nicotinic acid working through the high-affinity nicotinic acid G-protein-coupled receptor is particularly well suited for treatment of multiple sclerosis. The receptor is specifically expressed in one of the primary sources of MS pathogenesis (microglia). Furthermore this receptor is predicted to be involved in controlling the proliferation of autoreactive T cells via PGE_2_-mediated induction of IDO within the microglia (Figure 1). Nicotinic acid easily penetrates the blood brain barrier [44]. Nicotinic acid, but not nicotinamide, specifically binds to the high-affinity nicotinic acid G-protein-coupled receptor HM74a/GPR109a whose expression is largely restricted to professional antigen presenting cells including microglia. Since both nicotinamide and nicotinic acid contribute to greater NAD synthesis but only nicotinic acid signals through HM74a activation, we may consider nicotinamide in part a negative control for HM74a-mediated phenomena. Binding of nicotinic acid to HM74a leads to a massive release of prostaglandin PGD_2_ and PGE_2_ [45–47]. These prostaglandins then mediate the vasodilation that generates a flushing side effect [48]. PGD_2_ produced from nicotinic acid signaling then degrades to form 15d-PGJ_2_, which activates PPAR*γ* leading to increased expression of ABCA1 and CD36 in macrophages [28, 29]. The other prostaglandin PGE_2_ induces expression of IDO in dendritic cells, resulting in a toleragenic effects on local T cells [49, 50]. IDO serves specific functions in microglia [51–53], and IDO helps prevent EAE pathogenesis [52, 54]. Thus nicotinic acid is particularly wellsuited for consideration in the treatment of multiple sclerosis.

Cannabis-derived natural products including delta-9-tetrahydrocannabinol (Δ9-THC) also have a long history of significantly delaying the onset of EAE [57–59] and immune suppression in general [60]. The oromucosal spray known as Sativex contains these natural products (Δ9-THC and cannabidiol) and is currently used for treating the neuropathic pain and spasticity associated with MS [61, 62]. After the isolation of endogenous molecules that bind to the same G-protein coupled receptors as Δ9-THC, these “endocannabinoids” were also shown to provide relief from a viral-based animal model of MS, Theiler's Murine Encephalomyelitis Viral-immune demyelinating disease (TMEV-IDD; [63, 64]). However, only within the past several years has it become established that cannabinoids and endocannabinoids are in fact PPAR activators themselves [65, 66]. Significantly, in some cases PPARs are required to mediate their actions. This includes the anandamide-mediated PPAR*γ*-dependent therapeutically desirable repression of interleukin-2 (IL-2 [67, 68]), a recently identified MS disease susceptibility gene [69]. Given their ability to function as endogenous PPAR agonists combined with their potential value in autoimmune demyelinating disease, anandamide and 2-arachidonoyl glycerol should be examined with respect to their effects on gene expression related to MS pathogenesis.

## 2. The Gold Standard of Transcriptomic Modeling for Treating Multiple Sclerosis, Glucocorticoid Target Genes: IDO & IL-10

**Figure 2:**
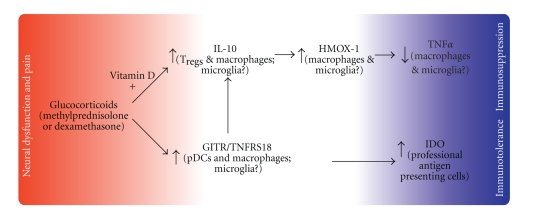
Glucocorticoids exert well-established relief from MS attacks, which last up to 30 days. Glucocorticoids exert immunoregulatory effects via transcriptional regulatory signaling in large part through GITR to IDO ([4, 5]) and IL-10 to HMOX-1 [8, 70, 71]). GITR activation is also known to lead to increased IL-10 production [72]. The representative glucocorticoid target genes of interest examined in this study with desirable effect are shown in bold. Multiple sclerosis patients are specifically lacking in CD46-stimulated IL-10 secretion and suffer from an over-proliferation of myelin autoreactive T cells.

Glucocorticoids comprise the most widely used drug class for providing rapid relief in acute attacks of MS. Thus, the immediate effect of glucocorticoids on target genes is the current gold standard for pharmacotherapeutic gene induction in MS treatment. Unfortunately, there is no reduction in relapse rate following glucocorticoid therapy, and relief is only temporary, lasting up to 30 days following a clinical attack. Given these limitations, there is an interest in sustained regulation of therapeutically beneficial glucocorticoid target genes by alternative approaches. Two recent studies suggest that both IL-10 and IDO likely mediate and determine the extent of glucocorticoid effects (Figure 2; IL-10 [6], IDO [4, 5]).

Recent studies in glucocorticoid-resistant asthma patients also implicate vitamin D as a factor in glucocorticoid effectiveness [6]. In these patients, the addition of dexamethasone does not cause an increase in IL-10 secretion from CD4+ cells. However, the addition of vitamin D3 with dexamethasone therapy in this population restores glucocorticoid-mediated induction of IL-10 [6]. The authors suggest that vitamin D can help restore glucocorticoid responsiveness. The combination of glucocorticoid with vitamin D is known to stimulate differentiation of CD4+ T cells to regulatory T cells (Treg) and causes substantial release of IL-10 (Figure 2, [73]). The role of vitamin D in preventing EAE models of MS is well established [74, 75], and there is a clear inverse correlation between vitamin D intake and MS occurrence (for a review see [76, 77]). These observations suggest the possibility of providing glucocorticoid therapy in conjunction with vitamin D to extend therapeutic effects. Furthermore, the results illustrate the importance of ensuring that beneficial nuclear receptor agonists are supplied in adequate concentrations to prevent a rate limiting reduction in therapeutic benefit during acute MS attacks.

It should also be mentioned that vitamin D has recently been determined to be an inhibitor of PARP-1 [78]. PARP-1 is the primary enzyme responsible for inflammation-induced depletion of cellular NAD (Figure 1). High-affinity PARP-1 inhibitors such as minocycline [79] have already proven beneficial in reducing clinical symptoms of MS in EAE models. Minocycline is currently being evaluated in the clinical treatment of MS [80–83]. PARP-1 appears to play an important role in MS pathogenesis.

Glucocorticoids strongly induce expression of TNFRSF18/glucocorticoid-induced TNFR-related (GITR) gene, which leads to induction of the enzyme controlling *de novo* NAD biosynthesis, indoleamine 2,3-dioxygenase (IDO). Most significantly the induction IDO activity is required for the full glucocorticoid anti-inflammatory effect [4, 5]. Inhibition of IDO activity exacerbates experimental autoimmune encephalomyelitis [52, 54]. All indications are that IDO induction may hinder autoimmune demyelination by starving autoreactive T cells of the essential amino acid tryptophan. Th1-derived cytokines tumor necrosis factor-*α* (TNF-*α*) or interferon-*γ* (IFN-*γ*) induces transcription and activates IDO thus functioning as a timed feedback mechanism for limiting cytokine-stimulated proliferation of autoreactive T cells (for a review see [84]). The full potential for pharmacological exploitation of tryptophan depletion to promote immunotolerance in autoimmune disease remains largely unaddressed [85].

## 3. Enzymatic Target Genes of Interest to MS

Regulation of immune function ultimately requires some kind of enzymatic biochemistry. Three particularly desirable drug-mediated target gene inductions of interest in MS are IDO, mitochondrial superoxide dismutase (SOD2), and heme oxygenase-1 (HMOX-1). Increased expression of IDO [52, 54], SOD2 [86], or HMOX-1 [8] has been shown to exert protection against EAE pathogenesis. Thus, we are interested in small molecules that affect transcription of these genes. Here three respective paragraphs are devoted to discussing why transcriptional induction of these genes is expected to provide benefit in MS therapy.

The tryptophan depleting enzyme indoleamine 2,3-dioxygenase (IDO) is also beneficial in EAE disease models of MS [52, 54], where IDO expression in microglia is tightly regulated by cytokines during inflammation [51–53]. In fact, IDO is centrally involved in nearly every examined autoimmune disease (for a review see [85]). As described above, IDO even appears central to the mechanism of glucocorticoid-mediated anti-inflammatory effects [4, 5]. IDO mediates the most dramatic example of immunotolerance, fetal acceptance. Accordingly, IDO over-expression also prevents both transplant rejection [87] and the lethality of graft versus host disease [88]. Sometimes, the mechanism of enzyme action in the liquid immune system resembles microbial competition (for a review [84]). Professional antigen presenting cells (macrophages, dendritic cells, and microglia) use IDO activation to deplete local extracellular tryptophan. The effect is autotoleragenic through tryptophan starvation of tryptophan-sensitive circulating T cells. Professional antigen presenting cells exploit this primitive biochemical competition for nutrients to control T cell proliferation. The simplicity of the mechanism makes IDO particularly attractive for pharmacological intervention [85].

New studies reveal that increased heme oxygenase-1 (HMOX-1) can dramatically reverse paralytic EAE and prevent relapse [9]. This is in agreement with previous pharmacological analysis in EAE [89]. Absence of HMOX-1 enhances demyelination in EAE, while induction of HMOX-1 with hemin or cobalt protoporphyrin can delay EAE onset. IL-10, a critical glucocorticoid target gene as described above, requires HMOX-1 activity to exert its immunosuppressive effects and is a known inducer of HMOX-1 [8]. Lastly, the end product of HMOX-1-catalyzed reaction, carbon monoxide, can itself mediate beneficial effects against EAE and is currently being considered as an MS therapeutic [9]. Heme oxygenase catalyzes the degradation of heme to produce iron, carbon monoxide, and biliverdin, the latter of which is subsequently degraded to produce the potent antioxidant bilirubin. HMOX-1 gene transcription is under the control of electrophile response elements that mediate extremely responsive transcriptional inducibility in response to oxidative stress ([90] and unpublished observations in zebrafish larvae). Consequently, HMOX-1 is often elevated in disease states as the body attempts to deal with oxidative pathosis.

A mere doubling of mitochondrial superoxide dismutase protein encoded by SOD2 via retroviral-mediated gene insertion strongly protects against EAE-mediated demyelination [86]. Increased oxidative stress, including decreases in superoxide dismutase, is a challenge in MS patients [91]. Amyotrophic lateral sclerosis (ALS) is a more severe CNS disease than MS, causing massive degeneration of motor neurons. SOD1 mutations are the only identified genetic link to familial amyotrophic lateral sclerosis [92]. Increased SOD2 is known to provide protection against mutated SOD1 neurotoxicity [93, 94]. Superoxide dismutase serves essential functions as an endogenous free-radical scavenging antioxidizing enzyme. Loss of function of mitochondrial superoxide dismutase 2 (SOD2) results in perinatal lethality [95], while loss of cytosolic superoxide dismutase 1 (SOD1) results in hepatocellular carcinoma in mice [96]. Given that mitochondria are a well-established Achilles' heel on the way to cell death, it is worth considering that increased SOD2 expression may delay cell death due to SOD1 mutations. Modest increases in SOD2 are also known to increase lifespan in metazoans, where the pathway is now believed to involve reduction in insulin signaling [97].

## 4. Common Pathways and Characteristics of Nonsteroidal Endogenous Antineuroinflammatory Molecules

The endogenous nonglucocorticoid molecules shown to provide protection against MS or animal models thereof (EAE or TMEV-IDD) have a distinguishing common set of characteristics. By “endogenous,” We are referring not only to those molecules which are synthesized endogenously but also the vitamins, which are endogenous in the sense that they must be present for survival regardless of the site of synthesis. This list includes nicotinamide [31], vitamin D [74, 75], retinoic acid [24], 15d-PGJ_2_ (12–16; [23–27]), and the endocannabinoids [63, 64] (Figure 1). Natural product cannabinoids have also been shown to ameliorate EAE progression [98], thus further supporting the notion for a potential role for endocannabinoids in preventing autoimmune-mediated demyelination.

First, these molecules generally function as transcription factor ligands, which work through peroxisome proliferator-activated receptor PPAR*α*, PPAR*γ*, or vitamin D receptors, all of which must heterodimerize with retinoid X receptor-alpha (RXR) to mediate transcriptional effects (Figure 1). Second, these molecules are antiangiogenic. Vascular endothelial growth factor (VEGF) levels are elevated in virtually all known autoimmune diseases, and reduction of VEGF production tends to minimize autoimmune disease pathogenesis (for a review [99]). This is predicted since autoimmune diseases are diseases of hyperproliferation, and similar to solid tumors, they respond favorably when treated with antiangiogenics as discussed above. Third, many of the molecules that are effective against EAE also correct dyslipidemia, often raising high-density lipoprotein (HDL) while lowering triglycerides and low-density lipoprotein (LDL). Nicotinic acid/niacin provides the greatest boost of HDL levels of any known molecule, regulates angiogenesis, and activates PPAR*γ* indirectly by producing 15d-PGJ_2_ via the high-affinity nicotinic acid G-protein-coupled receptor HM74a/GPR109a located on professional antigen presenting cells [28, 100]. However, nicotinic acid has not been examined in the context of MS therapy. Thus, additional consideration of nicotinic acid as a potential MS therapeutic is certainly warranted.

Virtually every endogenous molecule known to provide protection against animal models of MS is distinguished as also possessing antiangiogenic activity. This list of MS ameliorating antiangiogenic molecules includes vitamin D [101], 15d-PGD_2_ [102, 103], retinoic acid (effects on EAE [104–107]; effects on angiogenesis [108–110]), and anandamide [111]. We have observed that NAD precursors exert positive angiogenic activities in the developing zebrafish (data not shown). This is likely due to the proven role of the NAD-responsive enzyme SIRT-1 in stimulating growth phase angiogenesis [112]. However, nicotinic acid seems likely to potentially possess antiangiogenic activities stemming from PGD_2_ production within professional antigen presenting cells which are expected to be anatomically localized to sites of inflammation. Thus, nicotinic acid may exert effects on angiogenesis independent of the developmental context, instead being dependent on the cellular localization of the GPR109a-expressing prostaglandin-producing professional APCs.

## 5. Discussion

Greater emphasis should be placed on exploring combinatorial approaches to treating autoimmune demyelinating disease. Numerous studies have detected increased therapeutic effectiveness in a variety of disease contexts when simultaneously activating both subunits of the PPAR:RXR*α* heterodimer (Figure 1). Most significantly, this combination of 15d-PGJ_2_ with 9-*cis* retinoic acid exerts an additive effect in ameliorating EAE [24]. In vitro this mixture exerts a cooperative inhibition of microglial cell activation [113] and a cooperative antiproliferative effect on coronary artery smooth muscle cells [114]. The combination of PPAR*γ* ligands with 9-*cis* retinoic acid increases the killing of multiple myeloma cells [115] and cooperatively suppresses expression of ADAMTS4/Aggregecanase-1 [116], a protein stimulated by IL-1 that erodes articular cartilage in arthritic disease. This latter effect alone strongly suggests that the combination of 15d-PGJ_2_ and 9-*cis* retinoic acid may be particularly useful for the treatment of the autoimmune disease rheumatoid arthritis.

It seems most likely that additional activation of other nuclear receptors may exert a synergistic therapeutically beneficial effect in treating MS. For example, vitamin D or endocannabinoids may also exert cooperative effects. New studies indicate that both cannabinoid receptors CB_1_ and CB_2_ must be activated in order to stimulate myelination [117]. Extrapolation suggests that more than one molecule would be required for any potential recovery from MS neurodegeneration. Even glucocorticoids are not enough to prevent relapse rate. A teleost-based EAE model should be considered toward achieving higher throughput in an animal model for directly comparing the many small molecule permutations of potential drug combinations for their therapeutic value in treating MS.

Positron emission tomography studies performed in mice have shown that nicotinamide penetrates the mammalian blood-brain barrier [118]. High doses of oral nicotinic acid are well established in their ability to help treat the CNS disease schizophrenia [119–121]. Dramatic increases in NAD were detected in the spinal cord of mice subcutaneously injected with pharmacological doses of nicotinamide with concomitant profound protection against behavioral defects, demyelination, and death from EAE [31]. Thus drug delivery aspects and pharmacokinetics of oral nicotinic acid or nicotinamide for treating MS are not expected to be an issue.

Given that glucocorticoids such as methylprednisolone are universally accepted as the first approach for treating acute attacks of MS. These glucocorticoids may be considered as the current gold standard for attempts to model desirable ligand-induced transcriptomic effects to treat MS. Glucocorticoids work in large part through induction of IDO and IL-10, the latter of which exerts its effects in part via HMOX-1 induction [8]. Consistent with a critical role for these specific glucocorticoid-target genes in providing relief against MS, ectopic studies have revealed that small increases in gene expression of the enzymes HMOX-1 [9], IDO [52, 54], or the mitochondrial superoxide dismutase (SOD2; [86]) prevent or reverse autoimmune demyelination.

The endogenous PPAR ligand activators 15d-PGJ_2_ and anandamide prevent demyelination in animal models of MS. Nicotinic acid, while untested in EAE models, is unique because of its ability to stimulate production of prostaglandins 15d-PGJ_2_ and PGE_2_, the latter of which increases IDO expression [49, 50]. Additionally, pharmacological nicotinic acid by increasing NAD concentrations is expected to activate SIRT-1, which has a protective effect against neurodegeneration involving microglia [37, 122]. Experimentally, combinatorial activation of PPARs, retinoid X receptor, and/or vitamin D receptor generally provides additive benefit against EAE. A higher throughput teleost-based EAE model is needed to compare the various pharmacological permutations.

In summary, more emphasis should be placed on developing a nonsteroidal antineuroinflammatory cocktail for treating MS starting with pharmacological doses of nicotinic acid, 15d-PGJ_2_, nicotinamide, anandamide, vitamin D, and 9-*cis* retinoic acid toward extending the time frame of MS therapeutic benefit provided by glucocorticoids while minimizing dangerous side effects. Studies performed using animal models of MS indicate that there are multiple potentially rate-limiting factors controlling immune-mediated demyelinating disease progression. The range of possible nutritional and biochemical deficiencies in the etiology of MS, which may adversely affect the healing process, is complex. Thus it would be most prudent to consider combinatorial approaches as a means for providing the most reliable therapeutic treatment of multiple sclerosis.

## Abbreviations

15d-PGJ_2_: 15-deoxy-Δ^12,14^-prostaglandin J (2)
2-AG: 2-arachidonoylglycerol
Δ9-THC: Delta-9-tetrahydrocannabinol
ALS: Amyotrophic lateral sclerosis
CBs: Cannabinoids
CB_1_, CB_2_: Cannabinoid receptor 1 and 2
EAE: Experimental autoimmune encephalomyelitis
GITR: Glucocorticoid-induced TNFR-related gene/TNF receptor superfamily, member 18; TNFRS18
HDL: High-density lipoprotein
IDO: Indoleamine 2,3-dioxygenase
IFN-*γ*: Interferon gamma
IL-2Ra: Interleukin 2 receptor alpha
LXR: Liver X receptor
MOA: Mechanism of action
NAD: Nicotinamide adenine dinucleotide
PAPC: Professional antigen presenting cells (microglia, dendritic cells, macrophages, and B cells)
PGC1*α*: PPAR*γ* coactivator-1
PGE_2_: Prostaglandin E2
PPAR*α* and PPAR*γ*: Peroxisome proliferator-activated receptor *α* and *γ*
ROS: Reactive oxygen species
RXR: Retinoid X receptor
TMEV-IDD: Theiler’s Murine Encephalomyelitis
Virus: Induced Demyelinating Disease
T_h1_: T helper cells
T_reg_: Regulatory T cells
TNF-*α*: Tumor necrosis factor alpha
VDR: Vitamin D receptor
